# Feasibility testing of a standardised virtual clinic for follow-up of patients after hip and knee arthroplasty

**DOI:** 10.1308/rcsann.2021.0356

**Published:** 2022-08-17

**Authors:** NJ Preston, GA McHugh, EMA Hensor, AJ Grainger, PJ O’Connor, PG Conaghan, MH Stone, SR Kingsbury

**Affiliations:** ^1^ Leeds Institute of Rheumatic and Musculoskeletal Medicine, UK; ^2^ University of Leeds, UK; ^3^ Leeds Biomedical Research Centre, UK; ^4^ Leeds Teaching Hospitals NHS Trust, UK

**Keywords:** Virtual clinic, Arthroplasty

## Abstract

**Introduction:**

Over 200,000 hip and knee total joint arthroplasties (TJAs) are performed annually in England and Wales. UK guidelines recommend regular follow-up because missed early failure can result in complex revision surgery, which places additional burden on overstretched orthopaedic services. This study evaluated the feasibility and acceptability of an expert, consensus-based, standardised virtual clinic (VC) approach for TJA follow-up.

**Methods:**

Five UK secondary care orthopaedic centres implemented a standardised VC. Feedback was obtained through patient satisfaction questionnaires and telephone interviews with arthroplasty care practitioners. Key stakeholders subsequently attended an expert discussion forum to achieve consensus on the final VC format and to address obstacles identified during testing.

**Results:**

From 19 June 2018 to 11 December 2018, 561 TJA patients [mean age (SD) 70 (9.4) years, 57.8% female, 69.0% hip TJA, 1–28 years postsurgery (median 5 years)] completed a VC. Of these 561 patients, 82.2% were discharged without attending an outpatient appointment and 46 (8.8%) required early face-to-face consultant review. Patient satisfaction with the VC was high (156/188; 83.0%); over 70% of patients indicated a preference for the VC.

**Discussion:**

This feasibility study suggested significant resource savings, including time spent by consultant orthopaedic surgeons in outpatient clinics, hospital transport and an estimated saving of up to two-thirds of usual clinic-allotted time. The expert discussion forum provided helpful feedback for supporting more efficient implementation of the VC.

**Conclusions:**

A standardised VC is a feasible alternative to outpatient clinics for the follow-up of hip and knee TJA patients, and is acceptable to key stakeholders, including patients.

## Introduction

Hip and knee total joint arthroplasties (TJAs) are increasingly common surgeries performed in the UK. Although rare, TJA failure can occur, and in some cases is asymptomatic. Whereas guidelines recommend regular TJA follow-up,^1^ with over 200,000 TJAs now conducted annually the burden of maintaining traditional outpatient TJA follow-up is significant and growing.^2,3^ Some orthopaedic centres restrict TJA follow-up to the first year postsurgery or omit it entirely, accepting the risks to the patient and the costs and demands on orthopaedic centres of missing patients who require revision surgery.^4^

Outpatient reform is a priority for the NHS, highlighted by the Royal College of Physicians, the NHS 10-year plan and Getting it Right First Time.^5–7^ Both primary and secondary care were forced to rapidly implement remote consultations for most nonurgent appointments during the COVID-19 pandemic. This could pave the way for a permanent shift in the delivery of many NHS services, particularly using versions of ‘virtual clinics’ (VCs) using patient-reported outcome measures (PROMs) and standardised forms.^8,9^ We evaluated the feasibility and acceptability for patients and orthopaedic staff of implementing a standardised approach to a VC follow-up approach for the follow-up of patients with hip and knee TJA,^10^ and provide considerations for wider implementation in orthopaedic centres.

## Methods

### Setting

Five large orthopaedic centres participated in this study (see Table 1). Sites were approached using snowball sampling to provide a mix of regional teaching hospitals and nonacute outpatient clinics,^11^ with variation in their clinical administration and practice.

### Procedures

Each site used VC documentation consisting of a patient-completed questionnaire, standardised radiology report and clinical management algorithm.^10^ Sites were requested to use the VC on 100 consecutive patients due for TJA follow-up.

Sites could adapt the guidance provided for implementing the VC to account for local variations in clinical practice, but were not permitted to change the content of the VC documentation. Arthroplasty care practitioners (ACPs) implemented the VC at each site as usual care. Patients were sent the questionnaire to complete at home with a leaflet explaining the VC and an invitation to arrange an x-ray at a convenient x-ray department. Patients returned the questionnaire when attending for the x-ray. Site 3 (see Table 1) adapted the VC questionnaire, without changing its content, for completion on MyPathway,^12^ a system for coordination of orthopaedic care pathways using phones, tablets and personal computers. Patients had the option to download a hard copy, or request one from their ACP. Follow-up telephone interviews with ACPs enabled any problems with either the documentation, procedure or implementation to be discussed.

Following completion of the VC, patients were sent a patient satisfaction questionnaire consisting of four yes/no questions, and five Likert responses (using smiley faces). Patients could write comments in two ‘free text’ spaces to describe why they were dissatisfied with the VC and for patients to suggest improvements to the VC experience, respectively.

### Expert discussion forum

An expert discussion forum was held with the aim of achieving consensus from a range of experts on the final design of the VC, and for gathering their advice on implementation in UK clinical practice. Invitations were sent through relevant orthopaedic and radiology associations, to patients through our PPIE networks and NHS clinics and to health professionals from each of the five sites who had implemented the VC and those who had previously helped to develop it.^10^ ACPs from each participating site described benefits and challenges with the VC, and how they overcame any difficulties. The expert discussion closed with a debate and anonymous vote on each of six questions (given in Results) to support the study aims.

### Statistics

Statistical analyses were carried out with SPSS Statistics for Windows, version 22.0. Descriptive statistics are presented as arithmetic mean, standard deviation (SD) or median and interquartile range as appropriate, and absolute and relative frequencies as appropriate for demographic and categorical variables on the patient-reported questionnaire, standardised radiology report and patient satisfaction questionnaire. Outcomes of the VC are presented as absolute and relative frequencies.

Exploration of potential associations between VC item responses and patient satisfaction questionnaire feedback were decided a priori, but the association between satisfaction (with the joint) and a preference for face-to-face clinic appointments was requested by the expert discussion forum delegates. Chi-square test *p*-values were adjusted to account for multiple testing using a two-stage false-discovery-rate-controlling procedure,^13,14^ with alpha set at 0.05. These corrections were performed using R version 3.5.2 (package multtest v2.28.0).^15^

Feedback from interviews with the ACPs, from patient satisfaction questionnaires and from the expert discussion feedback was summarised according to key issues.

## Results

From 19 June 2018 to 11 December 2018, 561 TJA patients (42.2% male) aged between 28 and 92 years (mean (SD) age 70 (9.4) years) completed a VC (see Table 1). Some sites had lower numbers of patients than anticipated because of delays in obtaining approval from their Research & Innovation (R&I) departments or clinical managers. The shortfall was made up by other sites. Patients demographics were similar to national averages for hip and knee TJA patients.^2^ Length of time between surgery and follow-up ranged from 11 months to 28 years (mean (SD) 6 years 5 months (5 years 6 months); median 5 years 3 months). Overall, more patients were followed up for a total hip replacement (THA, 387/561, 69.0%) than a knee replacement (TKA, 174/561, 31.0%), although there was some variation across sites. Of 387 hip TJAs, there were 333 cemented acetabular components (86%), 54 uncemented acetabular components (14%), 315 cemented femoral components (81.4%) and 72 uncemented femoral components (18.6%).

**Table 1:** Summary of TJA participants by site, gender and type of TJA

	Total *n* (%)	Site 1 Large regional teaching hospital	Site 2 Large acute teaching hospital	Site 3 Large regional teaching hospital	Site 4 Nonacute outpatients community clinic	Site 5 Nonacute specialist hospital including orthopaedics
Total	561	161	160	72	148	20
Male*	207 (42.2)	71	51	28	51	6
Female*	284 (57.8)	67	89	37	81	10
Hip TJA	387 (69.0)	103 (64.0)	160 (100)	42 (58.3)	82 (55.4)	0 (0)
Cemented acetabular component	333	98	122	39	74	0
Uncemented acetabular component	54	4	38	3	9	0
Cemented femoral component	315	49	155	35	76	0
Uncemented femoral component	72	54	5	7	6	0
Knee TJA	174 (31.0)	59 (36)	0 (0)	30 (41.7)	65 (44.6)	20 (100)

TJA = total joint arthroplasty

*Gender not given for 70 participants

### Patient-reported questionnaire

A full breakdown of VC responses for all 561 patients is shown in Table 2. For example, pain (indicated on an 11-point numerical rating scale (NRS)) was reported by 159 patients (28%, NRS>0; hip patients 98/387, 25.3%; knee patients 61/174, 35.1%). Severe pain (NRS>7) was reported by 36 patients (6.4%; 23 hip patients, 5.9%; 13 knee patients, 7.4%). More patients reported pain when using stairs than any other activity (21.0%, *n*=118/561; hip patients *n*=73/384 (19.0%); knee patients 45/174 (25.9%)).

**Table 2:** Outcomes of VC documents and patient-reported satisfaction questionnaire

Patient-completed VC questionnaire			
	Proportion of Yes responses to Yes/No questions, *n*/*N* (%)^1^		
Pain (reported out of 10 on NRS)	Hip (*n*=387)	Knee (*n*=174)	
No pain	289 (74.7)	113 (64.9)	
Pain from 1–3 (%)	34 (8.8)	24 (13.8)	
Pain from 4–6 (%)	41 (10.6)	24 (13.8)	
Pain from 7–10 (%)	23 (5.9)	13 (7.4)	
Pain reported as:			
Using stairs	73/384 (19.0)	45/174 (26.4)	
Lying in bed	60/385 (15.6)	29/174 (16.7)	
Walking on level surface	59/383 (15.4)	30/174 (17.2)	
Inflammation	10/384 (2.6)	19/174 (10.9)	
General questions			
Does joint feel unstable?	39/358 (10.9)	25/163 (15.3)	
Has had to visit GP with joint problems?	9/357 (2.5)	5/162 (3.1)	
Satisfaction with TJA	*n*=354	*n*=164	
Very dissatisfied	5 (1.4)	5 (3.0)	
Moderately dissatisfied	19 (5.4)	5 (3.0)	
Ambivalent	26 (7.3)	16 (9.8)	
Moderately satisfied	53 (15.0)	47 (28.7)	
Very satisfied	251 (70.9)	91 (55.5)	
Mobility questions			
Has walking distance changed due to TJA?	46/363 (12.7)	24/165 (14.5)	
Has had to start using a mobility aid?	42/358 (11.7)	18/162 (11.1)	
Has had falls due to joint replacement?	12/364 (3.3)	9/163 (5.5)	
Activity questions			
Can still do work, jobs or chores?	316/349 (90.5)	148/165 (89.7)	
Can participate in favourite activities?	318/356 (89.3)	146/164 (89.0)	
**Standardised radiology report: items for which evidence was seen on x-ray**. Items all given in format ‘There is evidence/no evidence of … (Delete as necessary).’ Radiology report form was completed by ACPs.			
Cemented hip cup		Uncemented hip cup	
Cement bone interface lucency^1^	14	Migration or change cup	0
Cup migration orientation	9	Change position liner	0
Periprosthetic bone lucency	2	Ceramic liner fracture	0
Severe polyethylene wear	0	Periprosthetic bone lucency	0
Fixation screw fracture	0		
Cemented femoral component		Uncemented femoral component	
Periprosthetic bone loss^2^	8	Bone lucency	3
Periosteal reaction^3^	3	Periosteal reaction	1
Stem position	0	Periprosthetic bone loss	1
Cement fracture	0	Stem position	0
Periprosthetic fracture	0	Periprosthetic fracture	0
Knee joint replacement			
Periprosthetic lucency	13		
Component spacer position	2		
Periosteal reaction^4^	1		
Tibiofemoral joint space or displacement	1		
Periprosthetic fracture	0		
^1^ One referred for urgent follow-up, probably in combination with pain score. ^2^ One referred for urgent follow-up, probably in combination with pain score. ^3^ Two referred for urgent follow-up. ^4^ Referred for urgent follow-up.			
**Patient feedback about the VC**			
Patient satisfaction questionnaire (total questionnaires returned, *N*=189)^1^	Response to question=Yes		
Item (question)	Total, *n*/*N* (%)	Hip *N*=143 (37.5%)	Knee *N*=46 (25.3%)
Did questionnaire gather what you think are the most important details? (*n*=188)	176/188 (93.1)	136/143 (95.1)	40/45 (88.9)
Did VC questionnaire cause you any confusion? (*n*=189)	17/189 (9.0)	13/143 (9.1)	4/46 (8.7)
Did you receive a clinic outcome letter? (*n*=188)	144/188 (76.6)	109/142 (76.8)	35/46 (76.1)
Do you prefer virtual clinic to a face-to-face appointment? (*n*=182)	129/182 (70.9)	99/138 (70.7)	30/44 (71.4)
Give your level of satisfaction with the virtual clinic	(*n*=188)	(*n*=142)	(*n*=46)
Very dissatisfied	3 (1.6)	1 (0.7)	2 (4.3)
Moderately dissatisfied	8 (4.3)	6 (4.2)	2 (4.3)
Ambivalent	21 (11.2)	15 (10.6)	6 (13.0)
Moderately satisfied	55 (29.3)	42 (29.6)	13 (28.3)
Very satisfied	101 (53.7)	78 (54.9)	23 (50.0)
^1^Some items omitted by respondents			

ACP = arthroplasty care practitioners; GP = general practitioner; NRS = Numerical Rating Scale (11-point scale from 0–10); TJA = total joint arthroplasty; VC = virtual clinic

^1^Some patients did not respond to all items. Combinations of these omissions cause variations in *N*.

Satisfaction with TJAs was high (85.3% of patients), and was similar between hip and knee patients. Few patients (34/518, 6.6%) expressed dissatisfaction with their TJA.

### Radiology report

Problems on x-ray were identified for 54 (9.6%) patients, with 4 patients reported as having two problems. X-rays were examined at each site by ACPs using their usual means of x-ray review, but reported using the VC standardised x-ray report developed as part of the VC.^16^ No problems occurred in the 54 uncemented acetabular components but 25 problems were identified in the 333 cemented acetabular components. There were 5 problems identified in the 72 uncemented femoral components of the hip TJA compared with 11 in the 315 cemented femoral components.

### VC outcome decisions

**Figure 1:**
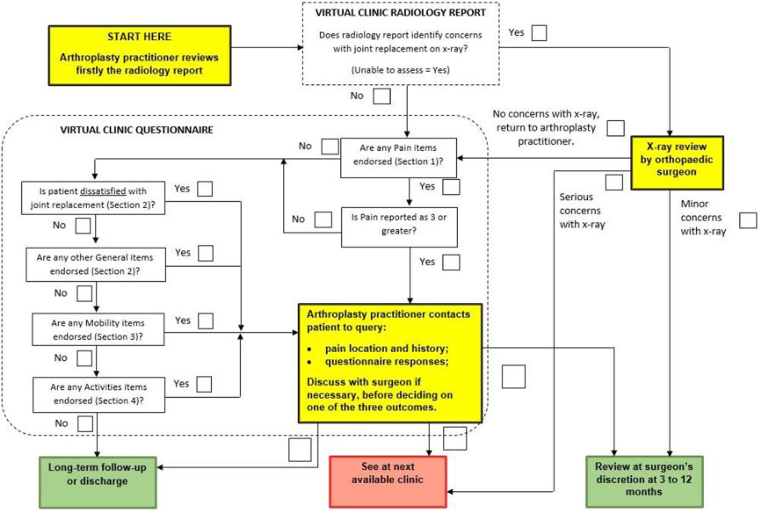
VC clinical decision algorithm. VC = virtual clinic

The VC decision algorithm (Figure 1) defines three possible outcomes, including long-term routine follow-up or discharge in line with British Orthopaedic Association (BOA) guidelines.^1^ In the 523 patients for whom the outcome was recorded, VC outcome decisions were similar for patients with hip and knee arthroplasty (Table 3). The majority of patients (hip: 308/369 (83.5%); knee: 122/154 (79.2%)) were either discharged or scheduled for long-term follow-up (eg, in 2 years), and 30/369 (8.1%) of patients with hip arthroplasty and 17/154 (11.0%) of patients with knee arthroplasty were listed as ‘review within 3–12 months’. This suggests that, in these patients, no problems were identified on x-ray and that either the patients indicated no problems on the questionnaire, or that any points raised on the questionnaire were addressed satisfactorily by a phone call from the ACP.

**Table 3:** Clinic outcomes by site (outcomes reported/total patients)

	Total	Site 1	Site 2	Site 3	Site 4	Site 5
	523/561*	156/161*	152/160*	66/72*	135/148*	14/20*
Discharge/long-term follow-up	430 (82.2)	119 (76.3)	134 (88.2)	49 (74.2)	115 (85.2)	13 (92.9)
Review within 3–12 months (at surgeon’s discretion)	47 (9.0)	37 (23.7)	0	4 (6.1)	6 (4.4)	0
See at next available clinic	46 (8.8)	0	18 (11.8)	13 (19.7)	14 (10.4)	1 (7.1)
*Outcome not recorded for all patients	38	5	8	6	13	6

Of the 523 patients, 46 (8.8%) were recalled for a consultation at the next available clinic (31/369 (8.4%)/15/154 (9.7%) of hip/knee patients, respectively). These appointments are initiated if (1) a problem is identified on the x-ray (note that this would be checked by the orthopaedic surgeon after being flagged by the ACP); (2) there is pain of 4/10 or greater (but only after symptoms are checked through a phone call by the ACP); or (3) any questionnaire items are endorsed (again, only after a phone call to check by the ACP).

### Expert discussion outcomes

Voting results of the expert discussion forum, feedback and considerations for implementation are presented in Tables 4 and 5. Important recommendations for improving communication with patients were noted and included in the ongoing use of the VCs at sites.

**Table 4:** Feedback on VC implementation and considerations for adoption in NHS practice

Feedback	Considerations for implementation	
*VC timings* ACPs reported that the new VC ranged from two minutes for patients reporting no problems to ten minutes for complex patients, and that it proved a useful training aid for ACPs. ACPs found the flexibility of the VC helpful; however, they emphasised that the VC requires allocated time in the working timetable, and that the time spent assessing VC patients (up to 25 per hour) is demanding and needs intense focus and concentration.	VC assessment should be scheduled into the clinic timetable.	
*Administration difficulties* ACPs reported occasionally forgetting to include the information leaflet with the clinic ‘appointment’ letter, causing problems such as patients arriving at outpatients departments for their ‘virtual’ clinic appointment, some having arranged hospital transport.	Communication with patients at time of surgery regarding likely VC follow-up mechanism would help to ensure that patients are not confused by the information received.	
*Urgent recall appointments* One unexpected problem reported by surgeons was the increased length of face-to-face orthopaedic clinic sessions (for patients who were recalled to be seen at the earliest opportunity). Recalled patients often presented with numerous potential areas of concern, causing each appointment to take longer than the standard clinic time. This had a knock on effect for the clinic as there were no ‘straightforward’ patients on the clinic list who help overrunning clinics to make up time.	Clinic schedules should be adapted to allow longer appointments for recalled patients.	
*Use of hospital resources* ACPs reported that, with fewer clinic appointments, use of hospital clinic facilities was greatly reduced, as were hospital transport costs, and the VC saved up to two thirds of usual clinic-allotted time. However, there was a significant increase in administrative burden for ACPs.	Employment of dedicated administrative assistants to support the delivery of VCs underpins their success. The increased cost of this administrative support is counteracted by savings in AHP and surgeon time, as well as hospital infrastructure costs.	
*Feedback on x-ray difficulties* Two sites reported difficulty with capacity in x-ray departments, and a third experienced an additional administrative burden by having to agree appointments for x-rays in advance on three days per week; in contrast, in clinics where patients were able to attend radiology on any day, within a window, and have an x-ray at their convenience, the VC worked more efficiently. Patients also reported increased satisfaction with this arrangement.	Radiology should consider whether flexibility can be provided for patients to attend and how they can encourage attendance at times when the service is less busy.	
*Potential problems of radiology report* Radiologists pointed out that surgeons and ACPs will need to take responsibility for identifying metastases, hernias and other problems, because the radiologists see only a small fraction of TJA x-rays. However it should be noted that the VC formalises and standardises a practice currently carried out across the UK and is no worse than the current outpatients system for follow-up of TJA in which x-rays are not reported on by radiologists. Nevertheless, the forum discussed a number of options designed to address this issue: • x-rays of the approximately 12% of patients not discharged by the VC could be sent for x-ray reporting by radiologists; • patients that report pain but have x-rays on which no problems are identified could have a second x-ray report, but completed by radiologists; • that the radiology report includes a check box to indicate that other radiological problems (‘incidental findings’) have not been identified.	Radiology reporting procedures and a system for checking and flagging other radiological problems should be considered and agreed at a local level. Flexibility is key.	
Radiologists further suggested inclusion of an item to check for loss of concentricity of acetabulum, which can be the only x-ray finding for cases of ceramic head failure. Ceramic head failure is known to be more common than ceramic liner fracture, another potential mode of failure and a radiologic red flag which was an item in the radiology report but which did not occur in the x-rays of any of the 561 patients.	This item and the checkbox will be included in future versions of the VC.	
*Patient feedback on satisfaction questionnaire* Two-thirds of patients who had expressed satisfaction with the clinic still expressed a preference for a face-to-face appointment. However, the results showed that patients who expressed preference for a face-to-face clinic had not received a clinic outcome letter, and half of the patients who received no feedback (22/44) about clinic outcomes left comments suggesting that this was a major problem with the VC.	A letter reporting joint status and clinic outcome should be generated for every patient and a copy sent to the patient as well as the GP	
Patients suggested that the questionnaire should ask about their other joints.	The covering letter should include reassurance that, if the patient has other TJAs, they will be evaluated in a separate future VC. In addition, the covering letter should state that if the patient was experiencing symptoms in a joint not previously referred for an orthopaedic opinion, the patient should visit their GP.	
Fourteen patients were confused about the VC, with some not understanding what one was or expressing confusion that they were not going to be seen for up to five years, or why they were not able to discuss their other joints. There were 28 patients who wanted to see a specialist under any circumstances, whether or not they were having difficulties or pain. Some patients complained of the impersonal nature of the VC, and the lack of opportunity to discuss other medical problems and medication.	A VC leaflet should be sent out with all clinic administration letters and the VC questionnaire. It should emphasise that this is about a single joint (and indicate which one); what the patient should do if they have any other joint problems or concerns that they wished to discuss with a medical professional; that testing of the VC shows that if the patient needs a face-to-face clinic appointment with a surgeon, the VC will identify this and will trigger that appointment.	
Patients appreciated the convenience of the VC, for example when attending for x-rays; some expressed satisfaction that it took less than one hour, and it had benefits for work and their family. However, for others, x-ray waiting times were a huge source of dissatisfaction, and they would have like to have been able to have their x-ray locally rather than at the central hospital.	See ‘Feedback on x-ray difficulties above.’ Flexible radiology appointments worked very well in those sites that implemented such a system, and were associated with higher satisfaction with the VC by patients	
Patients suggested that an online version of the questionnaire would have been welcome.	An online version of the questionnaire was successfully implemented at one site. However, this large city-based orthopaedic service found that many patients had no internet access and no plans to get online.	

ACP = arthroplasty care practitioner; TJA = total joint arthroplasty; VC = virtual clinic

**Table 5:** Results of voting by delegates attending the expert discussion forum

Attendees (19 experts): 1 programme leader for implementing an online patient outcomes capture system, 4 ACPs, 1 orthopaedic physiotherapist, 7 orthopaedic surgeons, 2 TJA patients and 4 radiologists		
Question	Voter options	*N* (%)*
This virtual clinic provides a solution to the problem of identifying the 5% of patients who need clinical follow-up following hip or knee arthroplasty while safely identifying those who are not at risk of revision.	Agree	19/19 (100)
	Disagree	0/19 (0)
Would you support the national roll out of this virtual clinic in its current form?	Yes	13/19 (68.4)
	No	5/19 (26.3)
If you said No to the previous question, what would improve your confidence in the virtual clinic? (Tick all that apply)	Additional items on the radiology report which I feel have been omitted.	10/18 (55.6)
	I support roll out of the virtual clinic..	8/18 (44)
	I would like to obtain endorsement of the appropriate national orthopaedic societies before supporting roll out of the virtual clinic.	8/18 (44.4)
	Additional items on the patient reported questionnaire, which I feel have been omitted.	6/18 (33.3)
	A follow-up after a year or more on all patients evaluated with the virtual clinic to confirm no adverse events contrary to virtual clinic findings.	4/18 (22.2)
	A service evaluation on more people who have had a knee or hip replacement	2/18 (11.1)
	Other (not captured)	1/18 (5.6)
As a patient, would you prefer this virtual clinic to a face-to-face appointment?	Note: two patients attended, and answered Yes. Four health professionals (one surgeon, one arthroplasty practitioner, one programme manager and one radiologist answered both as patients and health professionals)	Patient:
		Yes (6/18, 33.3%)
		No (0/18, 0%)
As an experienced health professional working in orthopaedic clinics, would you implement a post-TJA virtual clinic in your Trust if that virtual clinic met with your professional satisfaction?		Health professional:
		Yes (16/16, 100%)
		No (0/16, 0%)
If you cannot implement a virtual clinic, what prevents you?	I would implement this virtual clinic but include some adaptations for our particular clinical set up.	10/17 (58.8)
	Not enough administrative support.	8/17 (47.1)
	No adequate radiology support.	3/17 (17.6)
	I would implement this virtual clinic as it is.	3/17 (17.6)
	Leeds virtual clinic not adequately developed or demonstrated as effective.	2/17 (11.8)
	I believe in patient contact and face-to-face clinics	0/17 (0)

ACP = arthroplasty care practitioner; TJA = total joint arthroplasty

*19 delegates attended, but on questions 3 and 4, only 18 answered (one ACP missing responses), and on question 5, only 17 answered (one radiologist and one ACP missing responses).

### Feedback from patients using the VC

Of 561 patients, 189 (33.7%) returned VC satisfaction questionnaires, and their feedback and suggestions about the VC are shown in Table 4; 156/188 patients (83.0%) were satisfied with the VC, and this was similar for hip and knee patients. Seven (4.9%) hip patients and four (8.6%) knee patients were dissatisfied with the VC. Over 70% of patients (*n*=129) said that they would prefer a VC to a face-to-face appointment.

### Planned examination of associations between VC items and outcomes

Results of planned investigations into associations between VC items and/or patient satisfaction are listed in Table 6. There were associations between radiological problems and patients who reported falling, an unstable joint or joint pain.

**Table 6:** Associations between VC results

Associations between VC or patient satisfaction questionnaire results:	Likelihood ratio (95% confidence interval)	*p*-value*
No association between moderate to severe pain (greater than 3/10) and time since surgery (more than 7 years)	0.865 (0.608 to 1.232)	0.063
Patients were twice as likely to be satisfied with their TJA if they had pain of 3/10 or less	2.14 (1.7 to 2.7)	<0.001
Patients were 50% more likely to be satisfied with the VC if they had pain of 3/10 or less.	1.5 (1.2 to 2.1)	<0.001
Patients were twice as likely to have reported experiencing joint pain if there was a problem noted on the radiology report.	2.2 (1.2 to 3.9)	0.003
Patients were almost four and a half times more likely to have reported their joint feeling unstable if there was a problem noted on the radiology report.	4.4 (2.5 to 7.8)	<0.001
Patients were three and a half times more likely to have reported falling if there was a problem noted on the radiology report.	3.5 (1.5 to 8.1)	0.003
Patients reporting pain were more than twice as likely to express preference for a face-to-face clinic than those without any joint pain.	2.3 (1.5 to 3.5)	<0.001
If patients reported pain, those with 3/10 or less were 80% more likely to express a preference for a VC.	1.8 (1.2 to 2.8)	<0.001
Patients who did not receive a clinic outcome letter were twice as likely to report dissatisfaction with the VC.	2.0 (1.2 to 3.4)	0.005
Patients were 20% more likely to state a preference for a VC if they had received a clinic outcome letter.	1.20 (0.97 to 1.47)	0.044
Patients were over 60% less likely to state a preference for the VC if they were not discharged.	0.38 (0.20 to 0.71)	0.003
Patients were 73% less likely to express a preference for the VC if they had expressed dissatisfaction with their TJA.	0.27 (0.13 to 0.57)	<0.001

TJA = total joint arthroplasty; VC = virtual clinic

*p-values have been adjusted to control for multiple testing.^13,14^

Patients who reported dissatisfaction with the VC were more likely to have had pain, to have had concerns with other joints or had not received a letter with feedback from the VC review.

## Discussion

Information gathered from five diverse orthopaedic centres suggests that, logistically, the standardised approach for VC follow-up of TJA and management of patients ‘at risk’ could be integrated feasibly into routine clinical practice. One site showed that adaptation of the VC for online use to permit fully automated administration, with computerised preparation and printing of the clinic outcome letter, and thus reduced administrative workload, was also feasible. These benefits are similar to other online PROM capturing systems.^8^ Paper versions are likely to be necessary for the foreseeable future, as the site reported that 30% of its patients did not have online access and, in one of its local economically deprived satellite towns, 40% of patients had ‘no broadband or desire to get online’. However, research suggests that the older population are increasingly moving online and will adapt to new technology.^8^

Overall, 8.8% of patients for whom outcomes were recorded were recalled for a consultation at the next available clinic. This figure is lower than a previously published single-site study where 15% were recalled, and an Australian study of virtual hip and knee follow-up using a radiograph combined with an Oxford Hip or Knee Score rather than a consensus-designed tool, where 25% were recalled.^9,16^ However, it is still much higher than the cumulative percentage probability of revision at any of the time points for which this is recorded (0.78% and 0.47% for hips and knees, respectively, at 1 year, and 6.83% and 3.82% for hips and knees, respectively, at 13 years).^2^ There was a large difference between sites in the number of patients recalled for urgent follow-up. This could be explained by previous experience of sites using this VC, eg, site 1 had used a similar version and recalled no patients urgently, but did instead list at least four times as many patients for 3–12 month review.

Based solely on the algorithm, we identified 75% patients who met the algorithm criteria for discharge or future long-term follow-up, either immediately or following telephone review. In reality, 77% patients were discharged or placed on long-term follow-up, indicating that sites were following the algorithm to determine the ongoing management of patients reviewed through the VC. A 1-year follow-up audit of our previous single-centre VC study did not identify any patients that had re-presented with problems following discharge from the VC. Future work will audit long-term follow-up of patients reviewed through the standardised VC to ensure that patients with potential problems are not missed.

Most (13/19 68%) of the expert discussion forum attendees supported a national roll out of the VC, but 5 (26.3%) did not. One surgeon suggested that, for some orthopaedic centres, successful implementation of the VC will require a cultural change. However, he felt that his assessments and surgery would improve as he would be challenged more often by patients with complications requiring greater experience and skill while his juniors would deal with and benefit from more time with routine assessments and surgeries. Other surgeons recognised the potential pitfall of the VC for placing departments at a possible economic disadvantage, in that the VC could see orthopaedic teams seeing over 80% fewer TJA patients, with a corresponding loss of income per patient not physically seen in clinic. Six respondents suggested that important items were omitted, and that these need to be added. No suggestions were left as to what these items were, however, and we feel it is important to emphasise the extensive iterative process by which the questionnaire was developed by leading experts,^10^ both in the forum and from the important societies that are needed to give final approval. This paper is intended to be the first step towards acquiring this approval.

As with VCs in other medical specialities,^17–19^ the increased administrative burden was the largest difficulty, but this was resolved by dedicated administration support staff. The VC required adaptation of administration pathways, creation of new clinic codes and changes to Patient Administration System pathways.

Patients dissatisfied with the VC suggested that this dissatisfaction could usually be resolved with an information leaflet enclosed with the first ‘appointment’ letter, and by a clinic outcome letter.

As reported by clinicians in other new VCs,^18^ patients identified as having potential problems who are recalled for face-to-face appointments are patients with complex needs who take much longer to assess and consult. We recommend extending the length of appointments allocated to these patients.

We recognise that only five sites took part in the service evaluation. However, each orthopaedic centre across the UK differs in their clinical administration and practice, and the five sites ranged from community clinics to large teaching hospitals. We emphasise that, while the VC documents and algorithm have been developed using expert opinion from senior orthopaedic specialists and patients from across the UK, sites can adapt its implementation to fit with their site’s clinical practice.

## Conclusion

This feasibility study illustrated that a standardised VC is a feasible alternative to outpatient clinics for the follow-up of patients with hip and knee TJA, and is acceptable to key stakeholders. Orthopaedic centres presented solutions to minor difficulties identified when initially implementing the VC. VC users suggested significant benefits, including savings in resources (time spent by consultant orthopaedic surgeons in outpatient clinics, hospital transport and clinic space), convenience to patients and high patient satisfaction.

## Notes

Open Access This article is licensed under a Creative Commons Attribution 4.0 International License, which permits use, sharing, adaptation, distribution and reproduction in any medium or format, as long as you give appropriate credit to the original author(s) and the source, provide a link to the Creative Commons license, and indicate if changes were made. The images or other third party material in this article are included in the article's Creative Commons license, unless indicated otherwise in a credit line to the material. If material is not included in the article's Creative Commons license and your intended use is not permitted by statutory regulation or exceeds the permitted use, you will need to obtain permission directly from the copyright holder. To view a copy of this license, visit http://creativecommons.org/licenses/by/4.0/.
